# Quantifying neurodegeneration within subdivisions of core motor pathways in amyotrophic lateral sclerosis using diffusion MRI

**DOI:** 10.1007/s00415-025-12920-9

**Published:** 2025-02-19

**Authors:** Hannes Almgren, Colin J. Mahoney, William Huynh, Arkiev D’Souza, Sienna Berte, Jinglei Lv, Chenyu Wang, Matthew C. Kiernan, Fernando Calamante, Sicong Tu

**Affiliations:** 1 Brain & Mind Centre, The University of Sydney, Sydney, Australia; 2 School of Biomedical Engineering, Faculty of Engineering, The University of Sydney, Sydney, Australia; 3 Sydney Medical School, Faculty of Medicine and Health, The University of Sydney, Sydney, Australia; 4 Department of Neurology, Liverpool Hospital, Sydney, Australia; 5 Sydney Neuroimaging Analysis Centre, Sydney, Australia; 6 Neuroscience Research Australia, University of NSW, Sydney, Australia

**Keywords:** Amyotrophic lateral sclerosis, Motor neuron disease, Diffusion MRI, Corpus callosum, Corticospinal tract

## Abstract

**Background:**

Diffusion MRI is sensitive to white matter changes in amyotrophic lateral sclerosis (ALS). The current study aimed to establish disease profiles across core motor pathways, and their relevance to clinical progression in ALS.

**Methods:**

Sixty-five participants (ALS = 47; Control = 18) were recruited for the study. White matter integrity of motor, somatosensory, and premotor subdivisions within the corticospinal tract and corpus callosum were quantified by fibre density, fibre-bundle cross-section, structural connectivity, and fractional anisotropy. Analyses focused on identifying diffusion metrics and tract profiles sensitive to ALS pathology, and their association with clinical progression.

**Results:**

Reduced fibre density of the motor subdivision of the corpus callosum (CC) and corticospinal tract (CST) demonstrated best performance in classifying ALS from controls (area-under-curve: CC_motor_ = 0.81, CST_motor_ = 0.76). Significant reductions in fibre density (CC_motor_:* p* < 0.001*;* CST_motor_: *p* = 0.016), and structural connectivity (CC_motor_: p = 0.008; CST_somatosensory_: p = 0.012) indicated presence of ALS pathology. Reduced fibre density & cross-section significantly correlated with severity of functional impairment (ALSFRS-R; CC_motor_: *r* = 0.52*, p* = 0.019*;* CST_motor_: *r* = 0.59*, p* = 0.016). The largest effect sizes were generally found for motor and somatosensory subdivisions across both major white matter bundles.

**Conclusion:**

Current findings suggest that ALS does not uniformly impact the corticospinal tract and corpus callosum. There is a preferential disease profile of neurodegeneration mainly impacting primary motor fibres. Microstructural white matter abnormality indicated presence of ALS pathology while macrostructural white matter abnormality was associated with severity of functional impairment. Quantification of white matter abnormality in corticospinal tract and callosal subdivisions holds translational potential as an imaging biomarker for neurodegeneration in ALS.

**Supplementary Information:**

The online version contains supplementary material available at 10.1007/s00415-025-12920-9.

## Introduction

Structural abnormalities affecting the corticospinal tract (CST) and corpus callosum (CC) are a core MRI disease feature of ALS pathology [1]. CST hyperintensity on T2-weighted MRI scans, for instance, is considered a prominent radiological feature that supports a clinical diagnosis of ALS when present with clinical features indicating central involvement (*i.e.*, spasticity, brisk reflexes) [2]. Similarly, the CC shows increased intensity on FLAIR scans compared to healthy controls, and this intensity has also been shown to correlate with functional impairment [3]. Notably, classification modelling, considering only the diffusivity of the CST and CC, have demonstrated 80–90% accuracy in distinguishing classical ALS patients from disease mimics [4].

Diffusion MRI (dMRI) holds significant potential for translation as a clinical imaging modality to detect and monitor ALS motor pathology. Traditionally, diffusion-tensor imaging (DTI) metrics have been extensively used in clinical research, however, a known limitation since their inception has been limited biological interpretability due to an inability to model the complex nature of white-matter organisation. Increasingly, the normalisation of high-angular dMRI being acquired in standard clinical settings has allowed the application of more advanced WM modelling frameworks, such as fixels (*i.e.,* specific fibre bundles within a voxel) instead of voxels as grid elements, allowing more accurate modelling of the complexity of WM crossing fibres [5]. This framework overcomes the oversimplification exhibited by the DTI model and yields metrics that are more meaningfully interpretable in terms of changes in fibre density (FD), fibre-bundle cross-section (FC), and a combination of fibre density & cross-section (FDC) [6]. In neurodegenerative diseases, a decrease in FD primarily reflects axonal degeneration, while a decrease in FC is indicative of macroscopic brain atrophy [5]. These metrics have the potential to significantly advance precision of quantifying in-vivo neurodegeneration for clinical monitoring and could be used as secondary endpoints in prospective therapeutic trials. The usefulness of fixel-based analysis (FBA) in the context of ALS was initially shown by Raffelt et al. [7] who identified two clusters for which FD decreased significantly in motor neurone disease (MND) compared to healthy controls, namely a large proportion of the CST and the posterior body segment of the CC connecting bilateral primary motor cortices.

While structural abnormality along the CST and CC is a core WM signature of ALS, their fibre populations connect to diverse cortical regions, including the premotor, motor, and somatosensory cortices. Disease profiles of the integrity of tract subdivisions and their relevance to disease course remains unclear. Specifically, whether there is preferential degeneration of WM fibers innervating the primary motor cortex, relative to adjacent premotor/somatosensory fibers in the early stage of disease as proposed by pathological staging models [8–10]. In this study we quantified subdivision-specific WM integrity within the CST and CC tract bundles. We compared somatosensory, motor, and premotor subdivisions of the CST and CC using high angular resolution dMRI in combination with fixel-based metrics, DTI-based metrics, and structural connectivity estimates. Our analyses focused on (1) differences between ALS and controls, (2) associations with functional impairment scores and disease progression rate, (3) classification performance, and (4) characterising longitudinal changes.

## Methods

### Participants

Sixty-five study participants (ALS = 47, Control = 18) were prospectively recruited from the Forefront MND Clinic (Brain and Mind Centre, The University of Sydney), a specialist MND referral centre. A subsample of 7 ALS patients were scanned longitudinally at ~ 7 months (± 1.6 stdev) follow-up. The clinical diagnosis of ALS was made based on established international consensus criteria [11, 12]. All ALS participants presented with apparent sporadic classical forms of ALS without dementia [13, 14]. Patients with genetic mutations of ALS-related genes (*e.g.,* C9ORF72, SOD1) were excluded. Patients’ functional impairment was assessed using the revised ALS functional rating score (ALSFRS-R), a multi-domain rating scale monitoring severity of bulbar, motor, and respiratory impairment in patients with ALS [15] ranging from 0 to 48 with lower scores reflecting greater functional impairment. Disease progression rate (DPR) was calculated as the difference in ALSFRS-R score from maximum score, divided by disease duration in months. This study was approved by the University of Sydney ethics committee (2021/283). All participants provided written informed consent in accordance with the Declaration of Helsinki.

### MRI acquisition

All participants were scanned at the Brain and Mind Centre with a 3T GE Discovery MR750 scanner (GE Medical Systems, Milwaukee, WI), using a 32-channel head coil. 3D T1-weighted MRI scans were acquired with 204 coronal slices; TR/TE/TI = 6.2/2.3/500 ms; flip-angle = 12°; matrix size = 256 × 256; 1 mm isotropic voxels. Whole-brain dMRI data were acquired using 3 shells (25/40/75 directions for 700/1000/2800 s/mm^2^, respectively; TE/TR = 100/4000 ms; flip-angle = 90°; 2 mm isotropic voxels; multiband factor = 3). Eight inter-leaved b0 images without diffusion weighting were also acquired, as well as three reverse phase-encoded b0 images for distortion correction.

### MRI analyses

All analyses were performed in line with recent recommendations [5, 16]. A detailed schematic of our analysis pipeline is shown in Supplementary Fig. 1.

#### T1-weighted MRI pre-processing

Images were preprocessed using FastSurfer [17], which included brain-extraction and tissue segmentation. Resulting tissue segmentations were concatenated into a single 5-tissue type (5TT) image for anatomically constrained tractography (ACT) [18].

#### Diffusion-weighted MRI pre-processing

All dMRI analyses were performed using the MRtrix3 software library (version 3.0.2) [19]. Preprocessing included denoising, Gibbs unringing, bias field correction, susceptibility and eddy current distortion correction [20–25]. All scans were subsequently visually inspected for quality. Five subjects were excluded from subsequent analyses due to the presence of significant artifacts.

#### Fibre orientation distribution (FOD) estimation and tractography

FODs were computed as recommended in Dhollander et al. [5]. For fixel-based analyses, a group averaged FOD template was created (based on 15 representative subjects from each group) using a tissue-unbiased brain template [26]. Each subject’s intensity normalized FOD image was subsequently registered to the study-specific template*.* For tractography, 20 million probabilistic streamlines were generated using ACT [18].

#### Tract of Interest (TOI) tractography

**Fig. 1:**
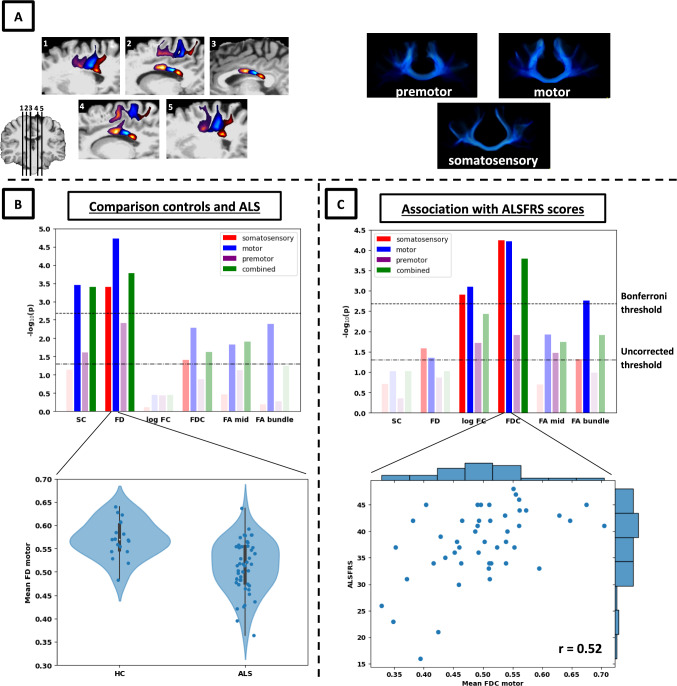
Cross-sectional results for the corpus callosum. Panel A shows the resulting track-density maps for sagittal slices (left; red = somatosensory, blue = motor, purple = premotor) and rendered image (right). Images were thresholded for illustrative purposes. Panel B shows the -log_10_ p-values for the comparison between ALS and controls (top) and details of largest effect in motor subdivision (bottom). Panel C shows the -log_10_ p-values for the association with ALSFRS-R scores (top) and an example of one of the largest effects in the motor subdivision (bottom; histograms in the margins show data distributions for the variables in the plot)

**Fig. 2:**
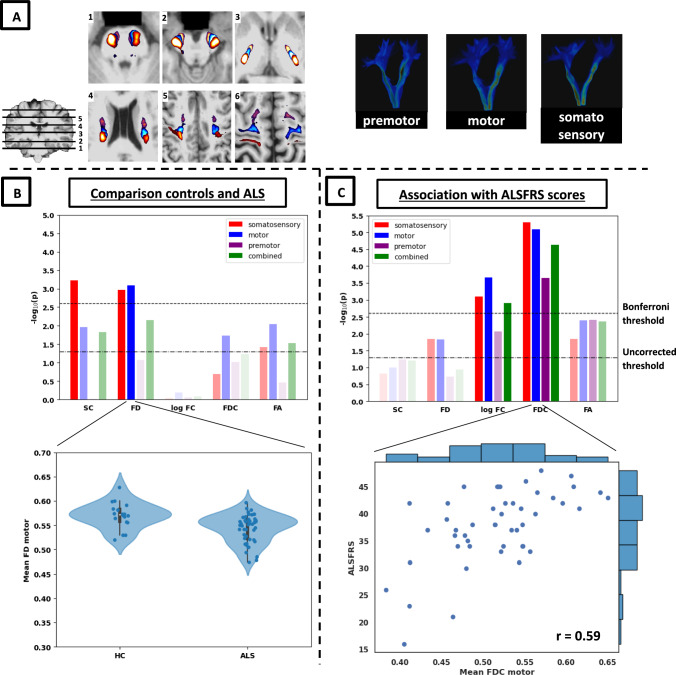
Cross-sectional results for the CST. Panel A shows the resulting track-density maps for axial slices (left; red = somatosensory, blue = motor, purple = premotor) and rendered image (right). Images were thresholded for illustrative purposes. Panels B shows the -log_10_ p-values for the comparison between ALS and controls (top) and details of largest effect in motor subdivision (bottom). Panel C shows the -log_10_ p-values for the association with ALSFRS.^6^-R scores (top) and an example of one of the largest effects in the motor subdivision (bottom; histograms in the margins show data distributions for the variables in the plot)

To segment the somatosensory, motor, and premotor subdivisions of CST and CC tract bundles, a recent digitized version of the Brodmann atlas was used [27]. This atlas was registered to subject-specific T1-weighted images for structural connectivity and diffusion tensor analyses, and to the T1-weighted group template for fixel-based analyses, using a combination of volume and surface-based registration [28, 29]. The somatosensory cortex was defined as Brodmann areas (BA) 1/2/3, primary motor cortex as BA4, and premotor cortex as BA6 [30, 31]. TOI subdivisions were analysed as independent within-tract components, and their combination as whole CST and CC tracts. Inclusion and exclusion regions-of-interest (ROIs) landmarks used to reconstruct CST and CC bundles are shown visually in Supplementary Figure 2. For the CST, tracking was performed between inclusion ROIs comprising cortical parcellations (somatosensory, motor, and premotor) and a manually delineated ROI at the level of the pons. Exclusion ROIs were generated encompassing the cerebellum and mid-sagittal CC. For the CC, identical cortical parcellations and mid-saggital CC masks were used as inclusion ROIs. Representative segmented subdivisions are shown in panel A of Figs 1 and 2.

#### DTI analysis

Track-density weighted-average DTI values [32] were computed for each TOI subdivision of the CST and CC (for an explanation of track-density imaging, see, [33]). Additionally, DTI values of the mid-sagittal CC ROI were calculated, where only a single fibre population is present and DTI provides a more biologically interpretable model. Our main DTI analysis focused on fractional anisotropy (FA). Results for mean diffusivity (MD), radial diffusivity (RD), and axial diffusivity (AD) are reported in Supplementary Figures 3 and 4.

#### Structural connectivity (SC) analysis

SC were computed from fibre-tracking using SIFT2-weights [16, 34] for each TOI subdivision of the CST and CC.

#### Fixel-based metrics

Fibre density (FD), fibre cross-section (FC), and fibre density & cross-section (FDC) metrics were calculated as outlined in Dhollander et al. [5]. Average FD, (log) FC, and FDC were computed for each TOI subdivision of the CST and CC.

### Statistical analyses

Data outside of the 1.75 × interquartile range was used as a conservative criterion to identify (and subsequently exclude) outliers for each metric (see, Supplementary Table 1). Three sets of permutation-based statistical tests and one ROC-curve analysis were carried out. First, we compared the average difference between healthy controls with ALS patients using linear models, with age as a covariate. Second, we assessed the association between ALSFRS-R total scores, DPR scores, and each diffusion metric using linear models, again controlling for age. DPR was log-transformed to minimize the influence of outliers. For each of these analyses, stringent Bonferroni corrections were applied across *all* metrics and TOI subdivisions, independently for the CST and CC, which was applied to avoid type-I errors (i.e., concluding there is an effect while there is none) and may differ from what has been done in other studies. Third, to assess each metric’s ability to categorize ALS from controls, we performed classification based on logistic regression models and receiver operating characteristic (ROC)-curve analyses were used to evaluate the model performance. The reported area under curve (AUC), sensitivity, specificity, and accuracy scores are the average values across 50 repetitions of stratified fivefold cross validation. Because of the imbalance in the number of controls compared to ALS, balanced accuracy scores were used (as implemented in scikit-learn). Finally, we assessed longitudinal changes in a subset of the ALS cohort. To have sufficient statistical power given our small longitudinal sample (N = 7), no multiple comparison correction was performed for the longitudinal analyses, which we restricted to the motor part of each tract to limit the number of comparisons. The longitudinal analyses are therefore highly exploratory. Longitudinal analyses on the remaining TOI subdivisions (*i.e.*, premotor and somatosensory) are reported in Supplementary Fig. 7.

Primary results for the permutation-based statistical tests are shown as negative log_10_ p-values to allow visual comparison of results for different diffusion metrics and to accentuate smaller p-values (*i.e.*, more significant results). To compare effect sizes between diffusion metrics more directly, Cohen’s *d* and Pearson’s *r* were calculated. TOI subdivisions are abbreviated in subscript after the tract name, for instance CC_ss_ depicts the somatosensory segment of the CC while CST_comb_ depicts the combined TOI subdivisions of the CST.

## Results

### Demographics and Clinical data

ALS and control participant cohorts did not significantly differ in terms of age or sex (Table 1; p-values > 0.10). The patient cohort was representative of classical ALS with 30% of patients presenting with an initial bulbar onset (n = 14). The longitudinal subset of ALS patients (n = 7) consisted of 4 males and 3 female patients, mean age of 62.9 years, and mean ALSFRS-R total score of 39.4.

**Table 1:** Mean and standard deviation (stdev). Disease duration was calculated from the date of initial symptom onset to the MRI scan date. A two-sample Welch’s t-test was performed for age and education. A chi-square test was performed for sex. ^1^Based on available data (36 ALS; 18 controls)

Variable	ALS (n = 47)	Control (n = 18)	p-value
Age (years)	61.2 (10.1)	63.9 (9.0)	*p* = 0.32
Sex	29 M/18F (61.7%)	7 M/11F (38.9%)	*p* = 0.10
Education (years)^1^	13.5 (3.3)	15.2 (2.8)	*p* = 0.08
ALSFRS (/48)	37.8 (6.9)	**–**	**–**
Disease Duration (months)	18.1 (13.2)	**–**	**-**
Disease Progression rate	(1.3)	**–**	**-**
Site of symptom onset	14 bulbar, 33 limb	**-**	**-**

### TOI Analyses

Fibre density and structural connectivity were significantly reduced in ALS compared to controls for both CC and CST (Figs. 1B, 2B), most notably for FD in the motor subdivision of the CC (CC_motor_: t = −4.72, *p*_*bonf*_ < 0.001). All significant decreases were found specifically in motor and somatosensory (but not premotor) subdivisions: in addition to the aforementioned decrease in the CC motor segment, FD was also decreased in the motor part of the CST (CST_motor_: t = −3.53, *p*_*bonf*_ = 0.016) and somatosensory segments of both bundles (CC_ss:_ t = −3.77, *p*_*bonf*_ = 0.01; CST_ss_: t = −3.43, *p*_*Bonf*_ = 0.022), while structural connectivity showed significant decreases in the motor segment of CC and the somatosensory segment of CST (CC_motor_: t = −3.86, *p*_*Bonf*_ = 0.008; CST_ss_: t = −3.63, *p*_*Bonf*_ = 0.012). Effect sizes (Cohen’s d) were on average 1.3 times larger for FD than for FA_bundle_ in motor subdivisions, and 1.1 times higher for SC than for FA_bundle_ in the same subdivisions (see, Table 2). Additional statistical details for the main diffusion metrics and for non-FA DTI metrics are shown in Supplementary Table 2 and Supplementary Fig. 3, respectively.

**Table 2:** Effect sizes for all diffusion metrics in the motor subdivisions of CC and CST

	Cohen’s *d*		Pearson’s *r* (ALSFRS)	
	CC_motor_	CST_motor_	CC_motor_	CST_motor_
SC	0.96	0.74	0.24	0.25
FD	**1.13**	**0.94**	0.29	0.36
log FC	−0.29	0.04	0.48	0.50
FDC	0.67	0.57	**0.52**	**0.59**
FA_mid_	0.72	n/a	0.37	n/a
FA_bundle_	0.79	0.76	0.45	0.42

Cohen’s *d* was used to compute differences between ALS and controls, while Pearson’s *r* was used to calculate associations with ALSFRS-R scores. Largest effect sizes for each segment are indicated in bold. *CC*  corpus callosum, *CST*  corticospinal tract, *SC*  structural connectivity, *FD*   fibre density, *FC*  fibre-bundle cross-section, *FDC*   fibre density & cross-section, *FA*   fractional anisotropy

### Clinical associations

This section is divided into two parts: (1) associations with ALSFRS-R and (2) associations with DPR.

#### Associations with ALSFRS-R

FDC and FC were positively associated with ALSFRS-R scores in both CC and CST (see, panel C of Figs. 1 and 2), indicating that higher white matter burden is related to increased level of functional motor impairment. FDC generally showed the largest associations, which were most notable in the motor and somatosensory segments for both the corticospinal tract (CST_motor_: *r* = 0.59, t = 5.13, p_*Bonf*_ < 0.001, CST_ss_: *r* = 0.60, t = 5.18, p_*Bonf*_ < 0.001) and corpus callosum (CC_motor_: *r* = 0.52, t = 4.39, p_*Bonf*_ = 0.001, CC_ss_: *r* = 0.54, t = 4.38, p_*Bonf*_ = 0.001). FDC in the combined bundles for both CST and CC was also positively related to ALSFRS scores (CST_comb_: *r* = 0.56; t = 4.66, p_*Bonf*_ < 0.001; CC_comb_: *r* = 0.49, t = 4.04, p_*Bonf*_ = 0.004). Finally, FC showed significant but consistently smaller associations with ALSFRS, primarily in motor and somatosensory segments of both bundles (CST_motor_: *r* = 0.50, t = 3.60, p_*Bonf*_ = 0.016; CST_ss_: *r* = 0.48, t = 4.05, p_*Bonf*_ = 0.004; CC_motor_: *r* = 0.48*,* t = 3.59, p_*Bonf*_ = 0.019; CC_ss_: *r* = 0.46, t = 3.46, p_*Bonf*_ = 0.030). Together, these results show higher effect sizes for FDC compared to FC, which were most notable for motor and somatosensory segments in both CC and CST. Correlations with FDC in the motor subdivisions were on average 1.3 times larger than for FA_bundle_, while correlations with (log) FC were on average 1.1 times larger than for FA_bundle_ (see, Table 2). Additional statistical details for the main diffusion metrics and non-FA DTI metrics are shown in Supplementary Table 3 and Supplementary Fig. 4, respectively.

#### Associations with disease progression rate (DPR)

We did not observe any significant associations with DPR that survived Bonferroni correction (see, Supplementary Fig. 5)***.*** At the *uncorrected* level, we observed associations with FA, most notably in the motor and premotor region of the CC (mid-sagittal ROI; CC_motor_: t = −3.15, p_*uncorr*_ = 0.003; CC_premotor_: t = −3.04, p_*uncorr*_ = 0.004) and with FDC in the somatosensory and motor subdivisions of the CST (CST_ss_: t = −2.11; *p*_*uncorr*_ = 0.04; CST_motor_: t = −2.05; *p*_*uncorr*_ = 0.05).

### Classification performance

**Fig. 3:**
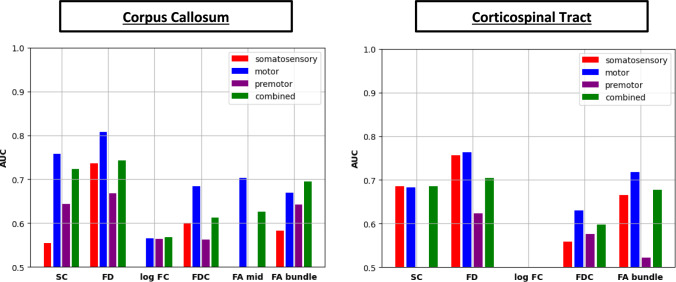
Results showing AUC values for CC (left plot) and CST (right plot). For displaying purposes, the vertical axes were truncated at 0.5

Results for the ROC-curve analyses (Fig. 3) showed that for both CC and CST, FD of the motor subdivision had the best overall classification performance (AUC/sensitivity/specificity/accuracy = 0.81/0.63/0.80/0.72; 0.76/0.60/0.74/0.67, for CC and CST respectively). In the CC, the second-best classification performance was SC of the motor subdivision (AUC/sensitivity/specificity/accuracy = 0.76/0.78/0.63/0.70), while in the CST, FD in the somatosensory subdivision showed the second-best classification performance (AUC/sensitivity/specificity/accuracy = 0.76/0.62/0.78/0.69). Overall, our classification results support our subdivision of the CC and CST into their motor and somatosensory subsegments. Sensitivity, specificity, and accuracy scores of all diffusion metrics are shown in Supplementary Fig. 6.

### Exploratory longitudinal results

**Fig. 4:**
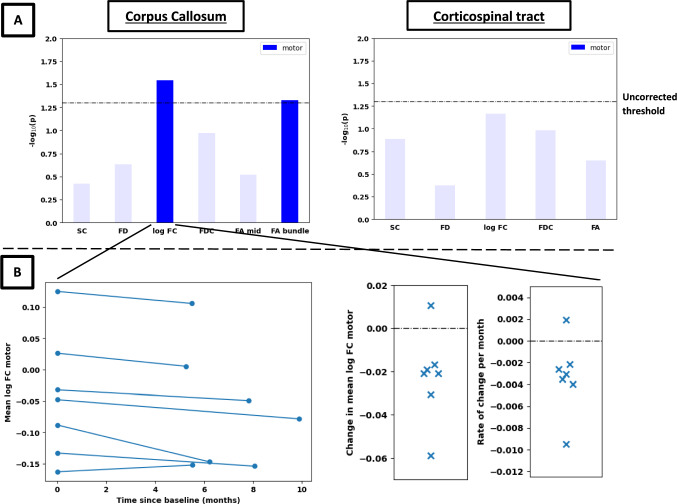
Longitudinal results for CC (top left plot) and CST bundles (top right plot). Panel A shows the negative log_10_ p-values, while panel B shows the longitudinal results for (log) FC in the motor subdivision of the corpus callosum for each individual subject

FC and FA_bundle_ of the CC showed a significant decline in the motor subdivision at follow-up compared to baseline (Fig. 4; t = −2.88, *p*_*uncorr*_ = 0.029; t = −2.47, *p*_*uncorr*_ = 0.047, respectively). We also found a significant longitudinal decrease in SC of the somatosensory part of the CST (t = −2.45, *p*_*uncorr*_ = 0.016; see Supplementary Fig. 7). Additional statistical details for the longitudinal analyses in the motor subdivision are shown in Supplementary Table 4.

## Discussion

The present study considered different subdivisions (motor, somatosensory, premotor) of the CC and CST and assessed the relevance of a variety of diffusion-based metrics to ALS diagnosis and severity of functional impairment. Our main results showed that motor and somatosensory subdivisions of both major fibre bundles show most marked effects of ALS with strongest associations with patients’ functional impairment, while premotor subdivisions generally showed smaller effects. Moreover, within fixel-based metrics we observed a distinct pattern: ALS showed specifically lower fibre density (FD) than controls, while functional impairment was inversely associated with fibre-bundle cross-section (FC) and a combination of fibre density & cross-section (FDC), likely reflecting a progression from microstructural axonal degeneration to macroscopic brain atrophy.

### In-vivo biomarkers of ALS and symptom severity

Our results suggest the presence of marked axonal degeneration associated with ALS pathology across core motor WM tracts, reflected by decreased FD especially in the motor cortex. There appears to be a notable dissociation, whereby functional impairment was selectively associated with reduced macroscopic WM integrity (*i.e.,* fibre-bundle cross-section), reflected in decreased FC and FDC, possibly a consequence of more severe axonal loss [5]. These results are in line with post-mortem and imaging studies showing reduced axon density in ALS [35], which over time may result in significant macroscopic brain atrophy [36]. This scenario also corresponds to the hypothetical scenarios outlined in Dhollander et al. [5] and Raffelt et al. [6], with the notable difference that in our case a decrease in FC was not accompanied by an increase in FD. Possibly, increases in FD are more detectable at later stages of the disease.

We did not observe significant associations between disease progression rate and diffusion metrics. There are a few possible reasons for the absence of this effect. First, the DPR scoring is partly based on subjective estimates of initial symptom onset, adding error-variance that could have obscured subtle associations with neuroimaging metrics. A more precise estimate of disease progression rate (*e.g.,* using longitudinal ALSFRS scores; [37]) could reveal more promising associations with diffusion-based metrics. Second, our analyses were based on cross-sectional imaging data which only provide a single snapshot of the disease. Longitudinal changes in diffusion metrics could be more related to the rate of disease progression, and predictive of future decline.

### Subdivision-specific profile of CST and CC in ALS

In general, we found that white matter abnormalities in ALS implicate motor and somatosensory subdivisions of major white matter bundles, which is in line with previous research [38]. This was confirmed by our ALSFRS analyses, showing that the strongest associations with somatosensory subdivisions were largely on par with motor subdivisions in both the CC and CST. Moreover, longitudinal analyses showed significant decreases over time, primarily in motor but also in somatosensory subdivisions (larger-scale longitudinal data should confirm these findings). In line with Braak’s staging model [8], these findings suggest that WM pathology in at least part of our sample had already spread to non-motor areas. Such spread of decreased WM integrity could be considered a biomarker of disease progression. Extensive longitudinal follow-up data would provide a more precise mapping of spread to nearby regions and other WM bundles.

### Evolving WM pathology

Our (exploratory) longitudinal analyses showed a decrease in FC and FA in the motor subdivision of the corpus callosum. The changes in FC suggest that more macroscopic changes such as brain atrophy happen throughout the course of the disease. Although we cannot separate out potential ageing effects given the limited sample size and absence of comparative longitudinal control data [39, 40], fibre-bundle cross-section in the corpus callosum does appear to be resilient to structural age-related degeneration across the lifespan [39].

### Fixel-based metrics as biomarkers of ALS

Fixel-based metrics generally outperformed DTI-based metrics in our sample in terms of (1) sensitivity to differences between ALS and controls, (2) performance in classifying ALS and controls, and (3) association with ALSFRS-R scores. This is consistent with other studies showing larger effects and increased sensitivity of fixel-based compared to DTI metrics, especially in areas with crossing fibers [5]. Fixel-based metrics are computed for more specific directions and have a higher biological interpretability in terms of specific underlying WM pathology. Therefore, these measures are more likely to capture purer bundle and disease-specific effects, especially in diseases such as ALS where specific fibre bundles are expected to be most affected. Raffelt et al. [41], for instance, showed increased sensitivity of FD compared to FA in detecting group-differences in MND, especially for the CST. Our study extended these findings to other fixel-based metrics (*i.e.,* FC and FDC), as well as to associations with functional scores in ALS. Interestingly, FA did show highest correlations with DPR (CC_motor_: *r* = −0.44), outperforming the FBA metrics (CC_motor_: r = −0.16 for FDC), however none of them reach statistical significance after Bonferroni correction.

### Clinical potential

Our results suggest that there is clinical potential for fixel-based metrics. Given their improved ability to distinguish between ALS and controls, fibre density (and to a lesser extent structural connectivity) has the potential to be used to better differentiate ALS from disease mimics [42, 43] and between ALS subtypes [44]. For instance, primary lateral sclerosis (PLS) has been found to show greater degeneration of precentral relative to postcentral gyri [45], while lower motor neuron impairment (*e.g.*, characteristic of progressive muscular atrophy) could demonstrate relatively more uniform WM degeneration of the proposed CST and CC subdivisions, which can both be tested with the proposed subdivisions. Fixel-based metrics also have the potential to be used as a tool for disease monitoring, as we found a significant longitudinal decrease in fibre-bundle cross-section, suggesting that this measure can pick up changes in white matter integrity. Given their larger sensitivity to ALS presence and symptom severity, fixel-based metrics also have the potential to be included in clinical trials as secondary outcome and therefore could decrease sample sizes needed to detect significant effects. Expanding our work to larger-scale longitudinal datasets, in combination with post-mortem data, could help towards the goal of using fixel-based metrics in clinical practice.

### Comparison previous FBA studies in ALS

Focusing on FD in the motor subdivision of the corticospinal tract, Nitert et al. [46] found that this metric discriminated well between ALS and controls, while it did not decrease significantly longitudinally *nor* was it associated with clinical upper motor neuron signs. Our results supplement their results by showing that it is mainly FC that decreases longitudinally and FDC that correlates well with clinical symptom severity. Cheng et al. [47] assessed fixel-based metrics in ALS for the corticospinal tract (not subdivided) and found significant differences with healthy controls for FD, FC, and FDC. Our results confirmed the between-group differences for FD, but we did not observe significant differences for FC (not even at the uncorrected level). This difference in results is likely explained by a difference in sample characteristics: our sample was less functionally impaired compared to the sample in Cheng et al. (average ALSFRS-R score = 37.8 versus 32.6, respectively). We showed that lower ALSFRS-R scores are related to lower FC, and therefore the difference in FC with controls would be smaller in our sample compared to their study. Contrary to our study, Cheng et al. [47] also observed a significant association between FD in the CST and ALSFRS scores. This difference between our studies could be a consequence of differences in statistical power, given that our correlation values for FD were within the same range. Their study had a slightly larger sample with relatively more functional impairment which could—at least partly—explain the difference in p-values. Interestingly, they did not observe a significant association between FC and ALSFRS-R, and therefore FD seemed to primarily drive the association with FDC. FC depends on image registration results [5], which in turn could depend on the b-value of the DWI sequence affecting the FOD template. Higher b-values, such as the ones used in our study, have been shown to be more optimal for fixel-based analyses [5]. Future larger multi-center studies could help explain the differences in FC findings between studies.

### Limitations and future directions

One of the limitations of the present study was the low number of ALS patients with longitudinal follow-ups and the absence of longitudinal control data. A larger longitudinal case–control sample with multiple follow-ups could give more conclusive evidence about the diffusion properties that change over time in ALS. Data from multiple sites may also need to be combined to achieve enough data. Furthermore, a larger control group may help reveal disease-specific effects and could more effectively exclude the effects of confounds such as sex (note that we did find similar results by analysing a subset of sex-balanced groups, suggesting our results hold irrespective of the influence of sex, see Supplementary Fig. 8). Another limitation relates to the brain parcellation. Our cortical parcellation was based on registration of a general Brodmann atlas to our individual T1-weighted images and group-template. Such combined volume-and-surface-based registration does not necessarily detect subject-specific subtle variations in cytoarchitectonic boundaries and therefore could have slightly obscured our effects. Higher-resolution data (*e.g.,* 7 T MRI) could potentially help in better detecting boundaries between different cortical regions. Furthermore, stronger gradients for diffusion MRI could improve tractography reconstruction and the estimation of several white matter characteristics [48]. Such data could also improve the segmentation of the CST into its motor/premotor/somatosensory parts, which showed quite some overlap *at the level of the brainstem* in our analyses (see the two most inferior slices in Fig. 2A), where CST is funnelled through a narrow brain area, and probabilistic tracking algorithms will naturally tend to create overlap. Note, however, that this overlap is reduced as the tract moved superiorly (see for example more superior slices in Fig. 2A) and completely disappear when the tracts terminate in the cortex (as the cortical ROIs were non-overlapping). Difficulties segmenting different parts of the CST have also been shown by He et al. [49]. Finally, post-mortem data could confirm the neurobiological interpretation of our findings. Future research could evaluate the usefulness of our subdivisions in distinguishing ALS from mimic diseases such as PLS [45].

## Conclusion

Our study showed that dividing the corpus callosum and corticospinal tracts into their motor and somatosensory subdivisions in combination with fixel-based metrics improves the sensitivity of diffusion MRI based markers in detecting disease-specific white matter alterations in ALS. Current findings support the presence of non-uniform profiles of axonal degeneration within primary motor tracts, selective to motor and somatosensory cortical motor neurons in the early stage of ALS pathology.

## Supplementary Information

Below is the link to the electronic supplementary material.

Supplementary file1 (PDF 684 KB)

Supplementary file2 (PDF 383 KB)

## Data Availability

The datasets generated during and/or analyzed during the current study are not publicly available due to sensitive patient information but are available from the corresponding author on reasonable request.

## References

[1] Tu S, Kiernan MC (2023) Chapter 14—Amyotrophic lateral sclerosis. In: Laule C, Port JD (eds) Advances in Magnetic Resonance Technology and Applications. Academic Press, pp 363–385

[2] Kiernan MC, Vucic S, Cheah BC, Turner MR, Eisen A, Hardiman O, Burrell JR, Zoing MC (2011) Amyotrophic lateral sclerosis. The Lancet 377(9769):942–955. 10.1016/S0140-6736(10)61156-7 10.1016/S0140-6736(10)61156-7 21296405

[3] Fabes J, Matthews L, Filippini N, Talbot K, Jenkinson M, Turner MR (2017) Quantitative FLAIR MRI in Amyotrophic Lateral Sclerosis. Acad Radiol 24(10):1187–1194. 10.1016/j.acra.2017.04.008 28572001 10.1016/j.acra.2017.04.008 PMC5605225

[4] Ferraro PM, Agosta F, Riva N, Copetti M, Spinelli EG, Falzone Y, Soraru G, Comi G, Chio A, Filippi M (2017) Multimodal structural MRI in the diagnosis of motor neuron diseases. Neuroimage Clin 16:240–247. 10.1016/j.nicl.2017.08.002 28794983 10.1016/j.nicl.2017.08.002 PMC5545829

[5] Dhollander T, Clemente A, Singh M, Boonstra F, Civier O, Duque JD, Egorova N, Enticott P, Fuelscher I, Gajamange S, Genc S, Gottlieb E, Hyde C, Imms P, Kelly C, Kirkovski M, Kolbe S, Liang X, Malhotra A, Caeyenberghs K (2021) Fixel-based analysis of diffusion MRI: methods, applications challenges and opportunities. Neuroimage 241:118417 34298083 10.1016/j.neuroimage.2021.118417

[6] Raffelt DA, Tournier J-D, Smith RE, Vaughan DN, Jackson G, Ridgway GR, Connelly A (2017) Investigating white matter fibre density and morphology using fixel-based analysis. Neuroimage 144:58–73. 10.1016/j.neuroimage.2016.09.029 27639350 10.1016/j.neuroimage.2016.09.029 PMC5182031

[7] Raffelt D, Tournier J-D, Rose S, Ridgway GR, Henderson R, Crozier S, Salvado O, Connelly A (2012) Apparent Fibre Density: A novel measure for the analysis of diffusion-weighted magnetic resonance images. Neuroimage 59(4):3976–3994. 10.1016/j.neuroimage.2011.10.045 22036682 10.1016/j.neuroimage.2011.10.045

[8] Braak H, Brettschneider J, Ludolph AC, Lee VM, Trojanowski JQ, Del Tredici K (2013) Amyotrophic lateral sclerosis—A model of corticofugal axonal spread. Nat Rev Neurol 9(12):708–714. 10.1038/nrneurol.2013.221 24217521 10.1038/nrneurol.2013.221 PMC3943211

[9] Eisen A, Braak H, Del Tredici K, Lemon R, Ludolph AC, Kiernan MC (2017) Cortical influences drive amyotrophic lateral sclerosis. J Neurol, Neurosurg Psychiatr 88(11):917–924. 10.1136/jnnp-2017-315573 10.1136/jnnp-2017-315573 28710326

[10] Eisen A, Kiernan M, Mitsumoto H, Swash M (2014) Amyotrophic lateral sclerosis: a long preclinical period. J Neurol, Neurosurg Psychiatr 85(11):1232–1238. 10.1136/jnnp-2013-307135 10.1136/jnnp-2013-307135 24648037

[11] de Carvalho M, Dengler R, Eisen A, England JD, Kaji R, Kimura J, Mills K, Mitsumoto H, Nodera H, Shefner J, Swash M (2008) Electrodiagnostic criteria for diagnosis of ALS. Clin Neurophysiol 119(3):497–503. 10.1016/j.clinph.2007.09.143 18164242 10.1016/j.clinph.2007.09.143

[12] Shefner JM, Al-Chalabi A, Baker MR, Cui LY, de Carvalho M, Eisen A, Grosskreutz J, Hardiman O, Henderson R, Matamala JM, Mitsumoto H, Paulus W, Simon N, Swash M, Talbot K, Turner MR, Ugawa Y, van den Berg LH, Verdugo R, Vucic S, Kaji R, Burke D, Kiernan MC (2020) A proposal for new diagnostic criteria for ALS. Clin Neurophysiol 131(8):1975–1978. 10.1016/j.clinph.2020.04.005 32387049 10.1016/j.clinph.2020.04.005

[13] Burrell JR, Halliday GM, Kril JJ, Ittner LM, Götz J, Kiernan MC, Hodges JR (2016) The frontotemporal dementia-motor neuron disease continuum. Lancet 388(10047):919–931. 10.1016/s0140-6736(16)00737-6 26987909 10.1016/S0140-6736(16)00737-6

[14] Kiernan MC, Vucic S, Talbot K, McDermott CJ, Hardiman O, Shefner JM, Al-Chalabi A, Huynh W, Cudkowicz M, Talman P, Van den Berg LH, Dharmadasa T, Wicks P, Reilly C, Turner MR (2021) Improving clinical trial outcomes in amyotrophic lateral sclerosis. Nat Rev Neurol 17(2):104–118. 10.1038/s41582-020-00434-z 33340024 10.1038/s41582-020-00434-z PMC7747476

[15] Cedarbaum JM, Stambler N, Malta E, Fuller C, Hilt D, Thurmond B, Nakanishi A (1999) The ALSFRS-R: A revised ALS functional rating scale that incorporates assessments of respiratory function. BDNF ALS Study Group (Phase III). J Neurological Sci 169(12):13–21 10.1016/s0022-510x(99)00210-5 10540002

[16] Smith R. E., Raffelt D., Tournier J.-D., Connelly A. (2022) Quantitative streamlines tractography: Methods and inter-subject normalisation. Aperture Neuro. 10.52294/ApertureNeuro.2022.2.NEOD9565

[17] Henschel L, Conjeti S, Estrada S, Diers K, Fischl B, Reuter M (2020) FastSurfer—A fast and accurate deep learning based neuroimaging pipeline. Neuroimage 219:117012. 10.1016/j.neuroimage.2020.117012 32526386 10.1016/j.neuroimage.2020.117012 PMC7898243

[18] Smith RE, Tournier J-D, Calamante F, Connelly A (2012) Anatomically-constrained tractography: Improved diffusion MRI streamlines tractography through effective use of anatomical information. Neuroimage 62(3):1924–1938. 10.1016/j.neuroimage.2012.06.005 22705374 10.1016/j.neuroimage.2012.06.005

[19] Tournier J-D, Smith R, Raffelt D, Tabbara R, Dhollander T, Pietsch M, Christiaens D, Jeurissen B, Yeh C-H, Connelly A (2019) MRtrix3: A fast, flexible and open software framework for medical image processing and visualisation. Neuroimage 202:116137. 10.1016/j.neuroimage.2019.116137 31473352 10.1016/j.neuroimage.2019.116137

[20] Andersson JLR, Skare S, Ashburner J (2003) How to correct susceptibility distortions in spin-echo echo-planar images: Application to diffusion tensor imaging. Neuroimage 20(2):870–888. 10.1016/S1053-8119(03)00336-7 14568458 10.1016/S1053-8119(03)00336-7

[21] Andersson JL, Sotiropoulos SN (2016) An integrated approach to correction for off-resonance effects and subject movement in diffusion MR imaging. Neuroimage 125:1063. 10.1016/j.neuroimage.2015.10.019 26481672 10.1016/j.neuroimage.2015.10.019 PMC4692656

[22] Cordero-Grande L, Christiaens D, Hutter J, Price AN, Hajnal JV (2019) Complex diffusion-weighted image estimation via matrix recovery under general noise models. Neuroimage 200:391–404. 10.1016/j.neuroimage.2019.06.039 31226495 10.1016/j.neuroimage.2019.06.039 PMC6711461

[23] Kellner E, Dhital B, Kiselev VG, Reisert M (2016) Gibbs-ringing artifact removal based on local subvoxel-shifts. Magn Reson Med 76(5):1574–1581. 10.1002/mrm.26054 26745823 10.1002/mrm.26054

[24] Skare S. & Bammer R (2010) Jacobian weighting of distortion corrected EPI data. Proceedings of the International Society for Magnetic Resonance in Medicine 5063

[25] Veraart J, Novikov DS, Christiaens D, Ades-aron B, Sijbers J, Fieremans E (2016) Denoising of diffusion MRI using random matrix theory. Neuroimage 142:394. 10.1016/j.neuroimage.2016.08.016 27523449 10.1016/j.neuroimage.2016.08.016 PMC5159209

[26] Lv J, Zeng R, Ho MP, D’Souza A, Calamante F (2023) Building a tissue-unbiased brain template of fiber orientation distribution and tractography with multimodal registration. Magn Reson Med 89(3):1207–1220. 10.1002/mrm.29496 36299169 10.1002/mrm.29496 PMC10952616

[27] Pijnenburg R, Scholtens LH, Ardesch DJ, de Lange SC, Wei Y, van den Heuvel MP (2021) Myelo- and cytoarchitectonic microstructural and functional human cortical atlases reconstructed in common MRI space. Neuroimage 239:118274. 10.1016/j.neuroimage.2021.118274 34146709 10.1016/j.neuroimage.2021.118274

[28] Postelnicu GM, Zöllei L, Fischl B (2009) Combined volumetric and surface registration. IEEE Transact Med Imaging (TMI) 28(4):508–522 10.1109/TMI.2008.2004426 PMC2761957 19273000

[29] Zöllei L, Stevens A, Huber K, Kakunoori S, Fischl B (2010) Improved tractography alignment using combined volumetric and surface registration. Neuroimage 51(1):206. 10.1016/j.neuroimage.2010.01.101 20153833 10.1016/j.neuroimage.2010.01.101 PMC2847021

[30] Geyer S, Schleicher A, Zilles K (1997) The somatosensory cortex of human: cytoarchitecture and regional distributions of receptor-binding sites. Neuroimage 6(1):27–45. 10.1006/nimg.1997.0271 9245653 10.1006/nimg.1997.0271

[31] Fulton JF (1935) A note on the definition of the “motor” and “premotor” areas. Brain 58(2):311–316. 10.1093/brain/58.2.311

[32] D’Souza A, Wang C, Tu S, Soligo DJ, Kiernan MC, Barnett M, Calamante F (2022) A robust framework for characterising diffusion metrics of the median and ulnar nerves: Exploiting state-of-the-art tracking methods. J Peripher Nerv Syst 27(1):67–83. 10.1111/jns.12478 34908209 10.1111/jns.12478

[33] Calamante F, Tournier J-D, Jackson GD, Connelly A (2010) Track-density imaging (TDI): Super-resolution white matter imaging using whole-brain track-density mapping. Neuroimage 53(4):1233–1243. 10.1016/j.neuroimage.2010.07.024 20643215 10.1016/j.neuroimage.2010.07.024

[34] Smith RE, Tournier J-D, Calamante F, Connelly A (2015) SIFT2: Enabling dense quantitative assessment of brain white matter connectivity using streamlines tractography. Neuroimage 119:338–351. 10.1016/j.neuroimage.2015.06.092 26163802 10.1016/j.neuroimage.2015.06.092

[35] Smith MC (1960) Nerve fibre degeneration in the brain in amyotrophic lateral sclerosis. J Neurol Neurosurg Psychiatr 23(4):269–282. 10.1136/jnnp.23.4.269 10.1136/jnnp.23.4.269 PMC497425 21610893

[36] Turner MR, Verstraete E (2015) What does imaging reveal about the pathology of amyotrophic lateral sclerosis? Curr Neurol Neurosci Rep 15(7):45. 10.1007/s11910-015-0569-6 26008817 10.1007/s11910-015-0569-6 PMC4443002

[37] Requardt MV, Görlich D, Grehl T, Boentert M (2021) Clinical determinants of disease progression in amyotrophic lateral sclerosis—A retrospective cohort study. J Clin Med 10(8):1623. 10.3390/jcm10081623 33921250 10.3390/jcm10081623 PMC8069893

[38] Tu S, Wang C, Menke RAL, Talbot K, Barnett M, Kiernan MC, Turner MR (2020) Regional callosal integrity and bilaterality of limb weakness in amyotrophic lateral sclerosis. Amyotrophic Lateral Sclerosis Frontotemporal Degeneration 21(5–6):396–402. 10.1080/21678421.2020.1733020 32106716 10.1080/21678421.2020.1733020

[39] Choy SW, Bagarinao E, Watanabe H, Ho ETW, Maesawa S, Mori D, Hara K, Kawabata K, Yoneyama N, Ohdake R, Imai K, Masuda M, Yokoi T, Ogura A, Taoka T, Koyama S, Tanabe HC, Katsuno M, Wakabayashi T, Sobue G (2020) Changes in white matter fiber density and morphology across the adult lifespan: A cross-sectional fixel-based analysis. Hum Brain Mapp 41(12):3198–3211 32304267 10.1002/hbm.25008 PMC7375080

[40] Kelley S, Plass J, Bender AR, Polk TA (2021) Age-related differences in white matter: understanding tensor-based results using fixel-based analysis. Cerebral Cortex (New York, NY) 31(8):3881–3898. 10.1093/cercor/bhab056 10.1093/cercor/bhab056 PMC8440891 33791797

[41] Raffelt DA, Smith RE, Ridgway GR, Tournier J-D, Vaughan DN, Rose S, Henderson R, Connelly A (2015) Connectivity-based fixel enhancement: Whole-brain statistical analysis of diffusion MRI measures in the presence of crossing fibres. Neuroimage 117:40–55. 10.1016/j.neuroimage.2015.05.039 26004503 10.1016/j.neuroimage.2015.05.039 PMC4528070

[42] Traynor BJ, Codd MB, Corr B, Forde C, Frost E, Hardiman O (2000) Amyotrophic lateral sclerosis mimic syndromes: a population-based study. Arch Neurol 57(1):109–113. 10.1001/archneur.57.1.109 10634456 10.1001/archneur.57.1.109

[43] Yedavalli VS, Patil A, Shah P (2018) Amyotrophic lateral sclerosis and its mimics/variants: a comprehensive review. J Clin Imaging Sci 8:53. 10.4103/jcis.JCIS_40_18 30652056 10.4103/jcis.JCIS_40_18 PMC6302559

[44] Swinnen B, Robberecht W (2014) The phenotypic variability of amyotrophic lateral sclerosis. Nat Rev Neurol 10(11):661–670. 10.1038/nrneurol.2014.184 25311585 10.1038/nrneurol.2014.184

[45] Finegan E, Chipika RH, Shing SLH, Doherty MA, Hengeveld JC, Vajda A, Donaghy C, McLaughlin RL, Pender N, Hardiman O, Bede P (2019) The clinical and radiological profile of primary lateral sclerosis: a population-based study. J Neurol 266(11):2718–2733. 10.1007/s00415-019-09473-z 31325016 10.1007/s00415-019-09473-z

[46] Nitert AD, Tan HH, Walhout R, Knijnenburg NL, van Es MA, Veldink JH, Hendrikse J, Westeneng H-J, van den Berg LH (2022) Sensitivity of brain MRI and neurological examination for detection of upper motor neurone degeneration in amyotrophic lateral sclerosis. J Neurol Neurosurg Psychiatr 93(1):82–92. 10.1136/jnnp-2021-327269 10.1136/jnnp-2021-327269 PMC8685620 34663622

[47] Cheng L, Tang X, Luo C, Liu D, Zhang Y, Zhang J (2020) Fiber-specific white matter reductions in amyotrophic lateral sclerosis. NeuroImage : Clinical 28:102516. 10.1016/j.nicl.2020.102516 33396003 10.1016/j.nicl.2020.102516 PMC7724379

[48] Jones DK, Alexander DC, Bowtell R, Cercignani M, Dell’Acqua F, McHugh DJ, Miller KL, Palombo M, Parker GJM, Rudrapatna US, Tax CMW (2018) Microstructural imaging of the human brain with a ‘super-scanner’: 10 key advantages of ultra-strong gradients for diffusion MRI. Neuroimage 182:8–38. 10.1016/j.neuroimage.2018.05.047 29793061 10.1016/j.neuroimage.2018.05.047

[49] He J, Zhang F, Pan Y, Feng Y, Rushmore J, Torio E, Rathi Y, Makris N, Kikinis R, Golby AJ, O’Donnell LJ (2023) Reconstructing the somatotopic organization of the corticospinal tract remains a challenge for modern tractography methods. Hum Brain Mapp 44(17):6055–6073. 10.1002/hbm.26497 37792280 10.1002/hbm.26497 PMC10619402

