# Supplementary material for: Quantifying neurodegeneration within subdivisions of core motor pathways in amyotrophic lateral sclerosis using diffusion MRI
Source: J Neurol. 2025 Feb 19;272(3):215. doi: 10.1007/s00415-025-12920-9 (PMC11839792; doi:10.1007/s00415-025-12920-9)
Supplement: Supplementary file 1 — Supplementary file1 (PDF 684 KB) [file 415_2025_12920_MOESM1_ESM.pdf]

Supplementary Figures

Supplementary Figure 1. Schematic overview of the diffusion MRI pipeline.

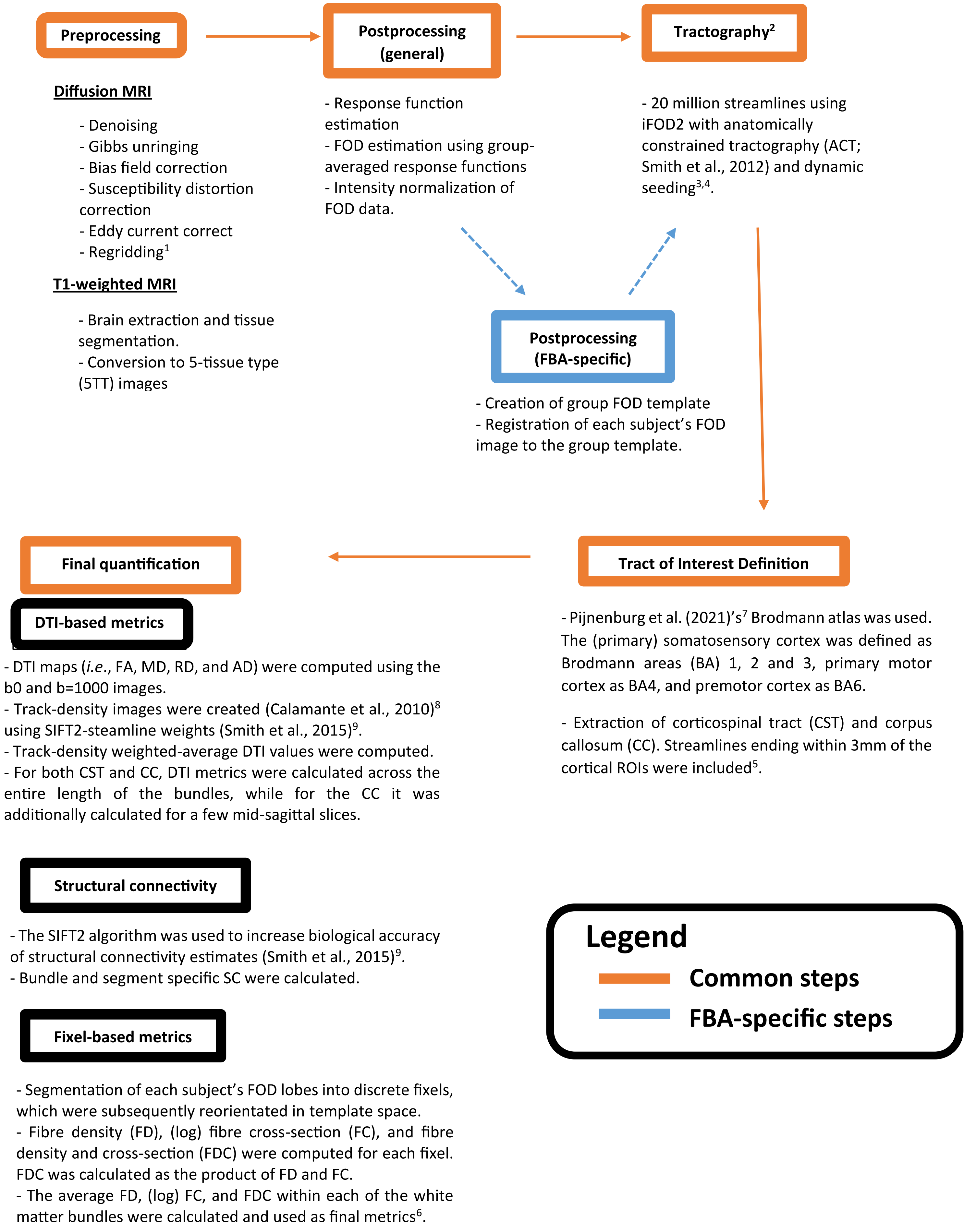

[1]1.25mm isotropic voxels size was chosen for fixel-based metrics based on the recommendations of the MRtrix3-pipeline (https://mrtrix.readthedocs.io/en/3.0.4/fixel_based_analysis/st_fibre_density_cross-section.html), while 1mm was chosen for DTI and SC based metrics.
[2]Performed on FOD-template for fixel-based metrics and in native subject-specific diffusion space for the other metrics.
[3]Other tracking parameters for FBA analyses were based on the suggested parameters in the MRtrix3-pipeline (see (1) above).
[4]To ensure reliable tracking of corpus callosum segments for each subject, additional streamlines were generated for subjects with below-median streamline count by seeding in each segment of the corpus callosum separately, until the median streamline count was reached.
[5]Note that each streamline was only assigned to one cortical ROI (the closest one) to avoid overlap between bundle endings. Streamlines near the edges were also only assigned to *either* the specified ROIs *or* the surrounding cortical regions (the latter streamlines were not used in our analyses).
[6]Due to the probabilistic nature of the tractography algorithm, we applied an empirically chosen threshold to the fixel TDI maps to exclude low density spurious tracts from FBA. Fixels that included less than approximately 0.2% and 0.05% of total streamlines were therefore excluded for the CC and CST, respectively.
[7]Pijnenburg, R., Scholtens, L. H., Ardesch, D. J., de Lange, S. C., Wei, Y., & van den Heuvel, M. P. (2021). Myelo- and cytoarchitectonic microstructural and functional human cortical atlases reconstructed in common MRI space. NeuroImage, 239, 118274. https://doi.org/10.1016/j.neuroimage.2021.118274
[8]Calamante, F., Tournier, J.-D., Jackson, G. D., & Connelly, A. (2010). Track-density imaging (TDI): Super-resolution white matter imaging using whole-brain track-density mapping. NeuroImage, 53(4), 1233–1243. https://doi.org/10.1016/j.neuroimage.2010.07.024
[9]Smith, R. E., Tournier, J.-D., Calamante, F., & Connelly, A. (2015). SIFT2: Enabling dense quantitative assessment of brain white matter connectivity using streamlines tractography. NeuroImage, 119, 338–351. https://doi.org/10.1016/j.neuroimage.2015.06.092

Supplementary Figure 2. Inclusion and exclusion ROIs to define the bundles:

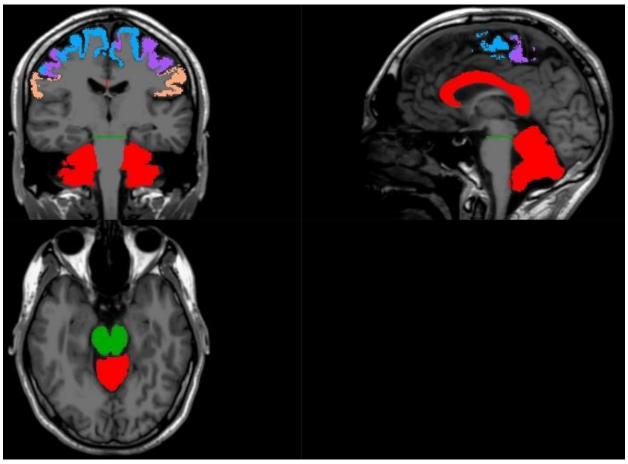

Supplementary Figure 2a. Cortical, inclusion and exclusion regions of interest (ROIs) for the CST. Cortical ROIs: somatosensory (pink), motor (purple), and premotor (blue) cortex. Inclusion ROI: supra-pons (green). Exclusion ROIs: cerebellum and mid-sagittal corpus callosum (red). Streamlines were only accepted if they ended within three millimetres from the cortical ROIs, traverse the inclusion ROI, and did not traverse or enter the exclusion ROIs.

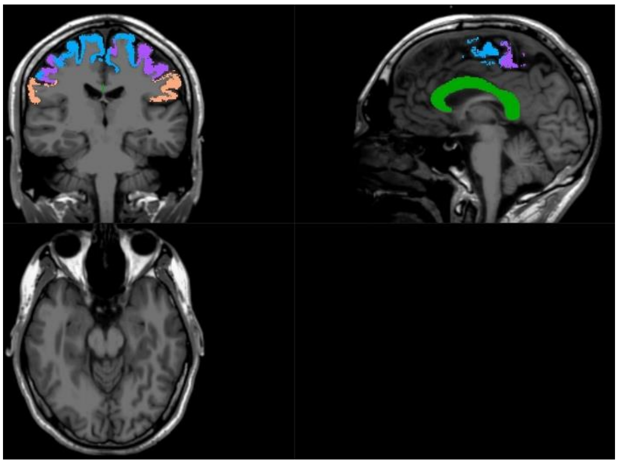

Supplementary Figure 2b. Cortical and inclusion ROIs for the CC. Cortical ROIs: somatosensory (pink), motor (purple), and premotor (blue) cortex. Inclusion ROI: mid-sagittal corpus callosum (green). Streamlines were only accepted if they ended at both sides (left and right hemisphere) within three millimetres from the cortical ROIs and traversed the corpus callosum.

Supplementary Figure 3. Differences ALS versus controls for non-FA DTI metrics:

Note that for consistency with the main analyses, the same number of comparisons are taken into account here, which was 24 for corpus callosum analyses, and 20 for CST analyses.

Supplementary Figure 3a: CC.

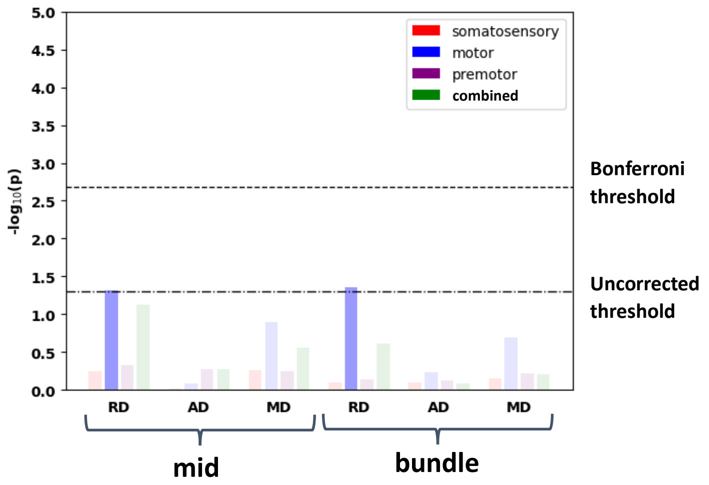

Figure 3a. Cross-sectional results for the corpus callosum, showing the -log10 p-values for the comparison between ALS and controls.

Supplementary Figure 3b: CST.

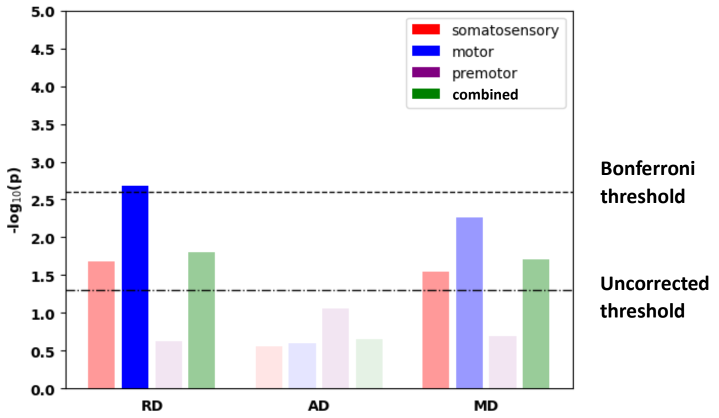

Figure 3b. Cross-sectional results for the CST, showing the -log10 p-values for the comparison between ALS and controls.

Supplementary Figure 4. ALSFRS-associations with non-FA DTI metrics:

Note that for consistency with the main analyses, the same number of comparisons are taken into account here, which was 24 for corpus callosum analyses, and 20 for CST analyses.

Supplementary Figure 4a. CC:

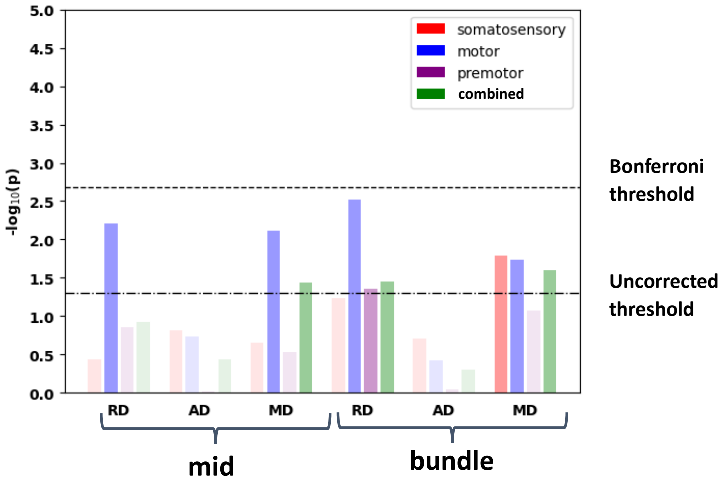

Figure 4a. Cross-sectional results for the corpus callosum, showing the -log10 p-values for the association with ALSFRS-R scores.

Supplementary Figure 4b. CST:

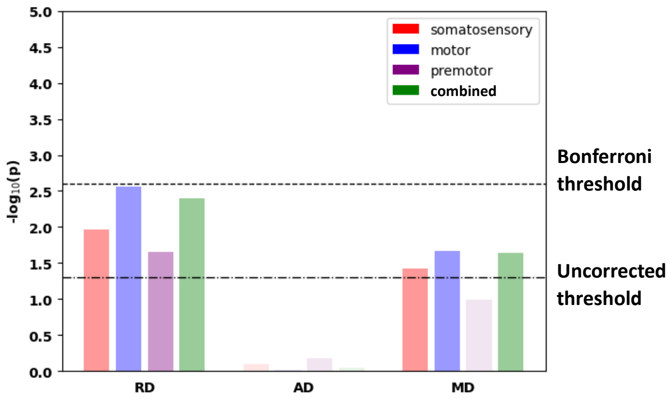

Figure 4b. Cross-sectional results for the CST, showing the -log10 p-values for the association with ALSFRS-R scores.

Supplementary Figure 5: Associations with disease progression rate (DPR)

Supplementary Figure 5a: CC.

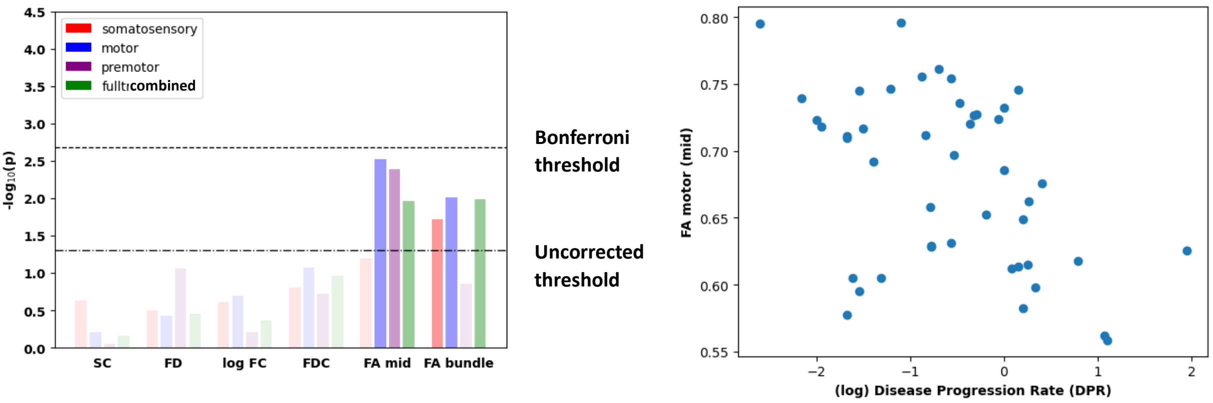

Figure 5a. Cross-sectional results for the corpus callosum showing the -log10 p-values for the association with (log) DPR scores (left) and a scatterplot of the association between FA-mid (motor) and (log) DPR (right).

Supplementary Figure 5b: CST.

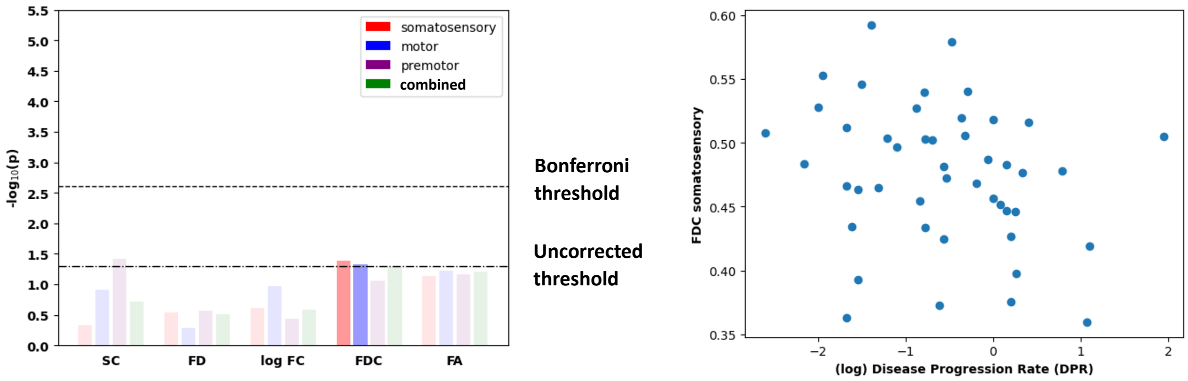

Figure 5b. Cross-sectional results for the CST, showing the -log10 p-values for the association with (log) DPR scores (left) and a scatterplot of the association between FDC (somatosensory) and (log) DPR (right).

Supplementary Figure 6. Sensitivity and specificity

Using minimal distance thresholding (lowest Euclidian distance ROC curve to upper left corner):

6a. sensitivity

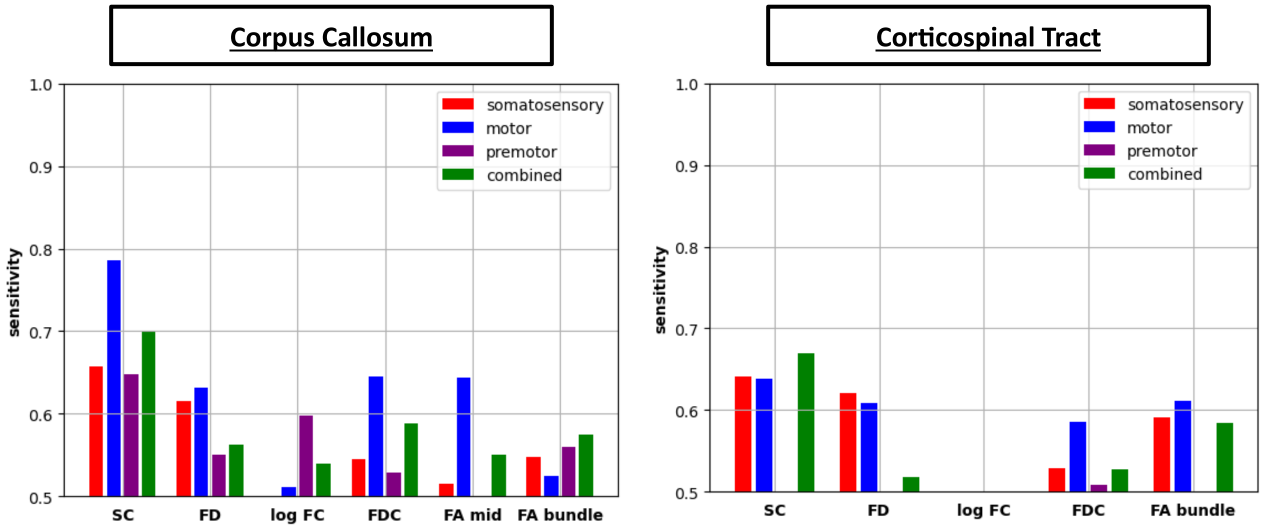

Figure 6a. Results showing sensitivity for CC (left) and CST (right). For display purposes, the vertical axes were truncated at 0.5.

6b. specificity

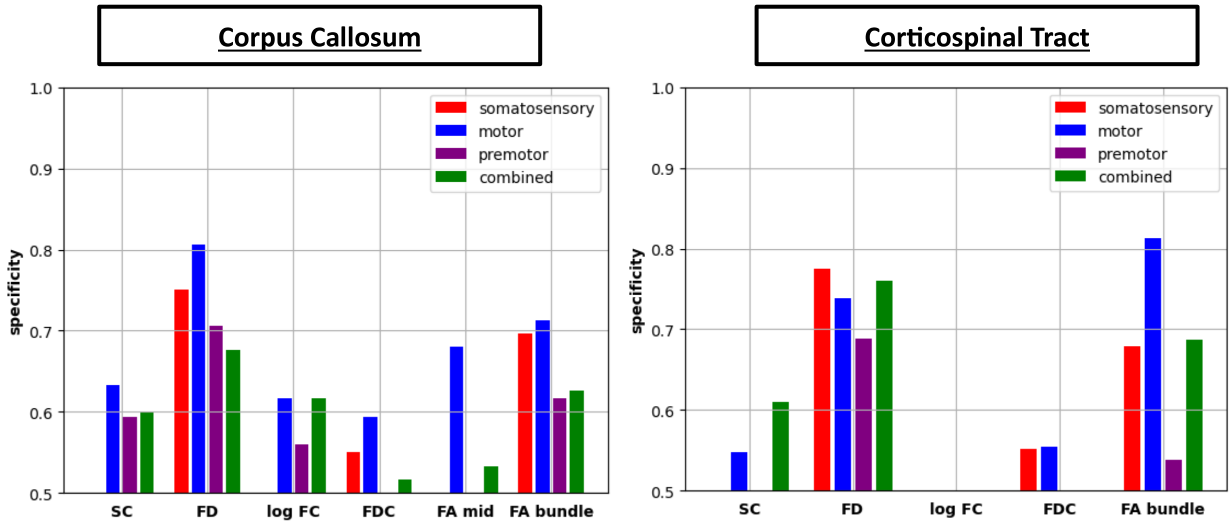

Figure 6b. Results showing specificity for CC (left) and CST (right). For display purposes, the vertical axes were truncated at 0.5.

6c. accuracy

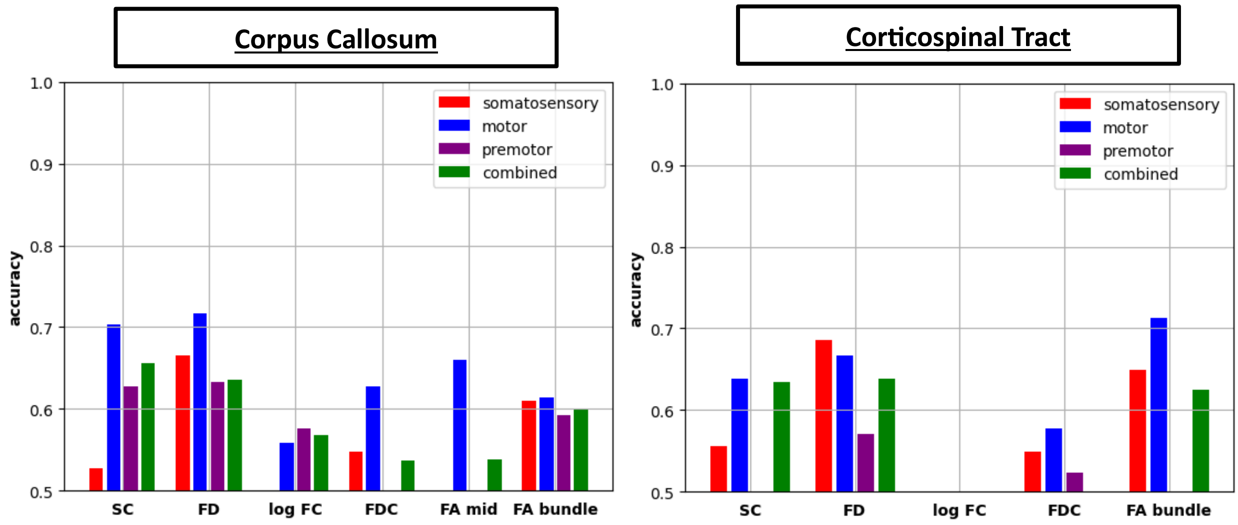

Figure 6c. Results showing accuracy for CC (left) and CST (right). For display purposes, the vertical axes were truncated at 0.5.

Supplementary Figure 7: longitudinal results for non-motor segments

7a. Corpus callosum:

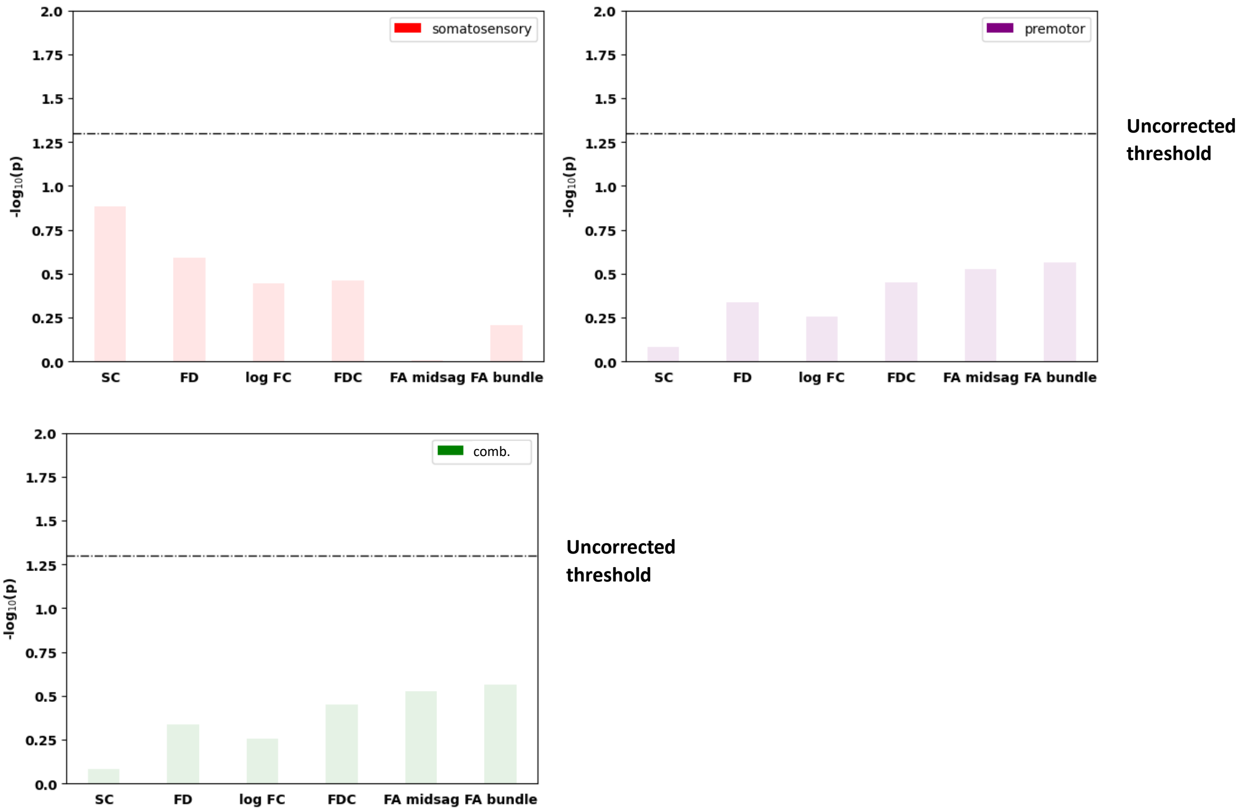

Figure 7a. Longitudinal results for corpus callosum bundles (showing negative log10 p-values) for the somatosensory segment (red), premotor segment (purple), and combined tract (green).

7b. CST:

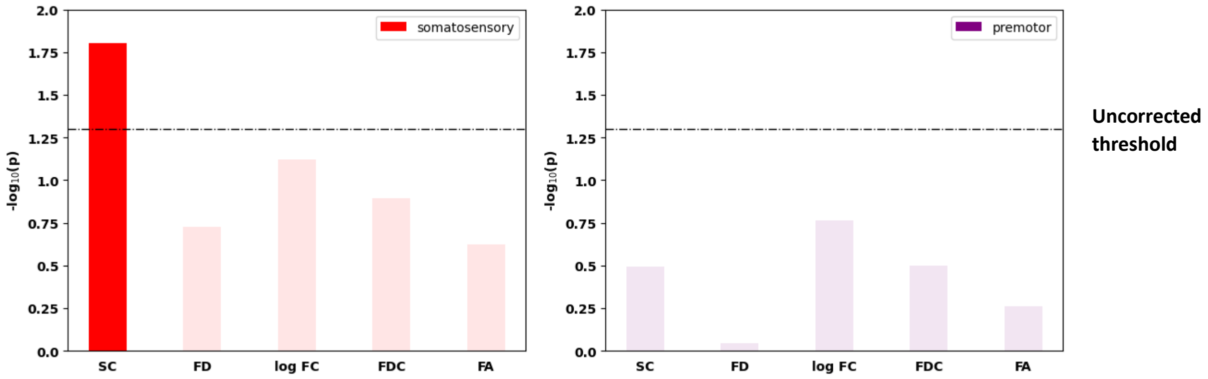

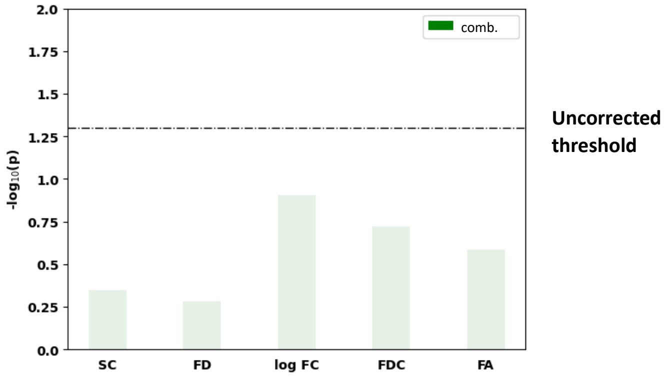

Figure 7b. Longitudinal results for CST bundles (showing negative log10 p-values) for the somatosensory segment (red), premotor segment (purple), and combined tract (green).

7c. Details significant results: decreased SC in somatosensory CST segment

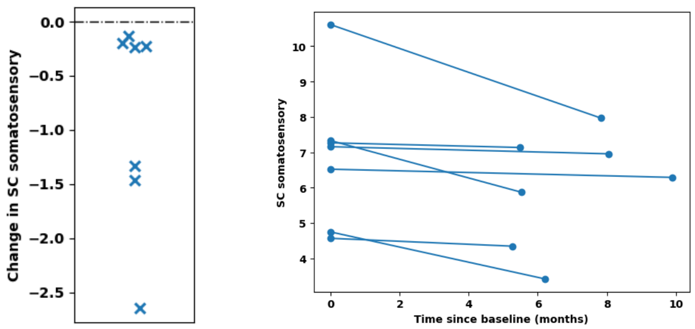

Figure 7c. Longitudinal results for SC of the somatosensory segment of the CST.

Supplementary Figure 8. Control analyses for subset gender-matched groups

Methods: In the main manuscript, sex was not corrected for in the analysis because of the low number of control samples, which could render the estimation of sex effects unreliable. However, because of the notable difference in sex composition, we checked consistency of our results with a subset sex-matched control group, dropping 18 male ALS patients at random. The average log p-values were calculated for 50 repetitions of exclusions, and these results were qualitatively compared to our original results.

8a. Corpus callosum:

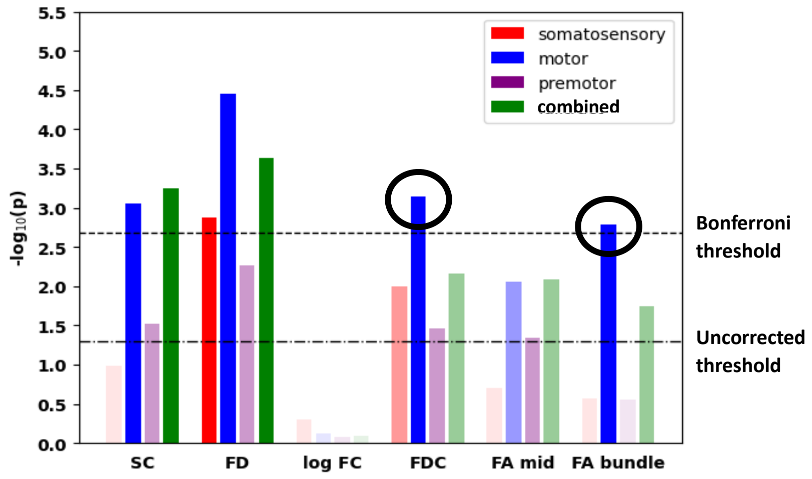

Supplementary Figure 8a. Analyses with matched-sex samples showed similar results (cf. Figure 1B), however in this comparison FDC and FA of the motor tract (indicated by black circles) also differed significantly between groups albeit less pronounced compared to FD.

8b. CST:

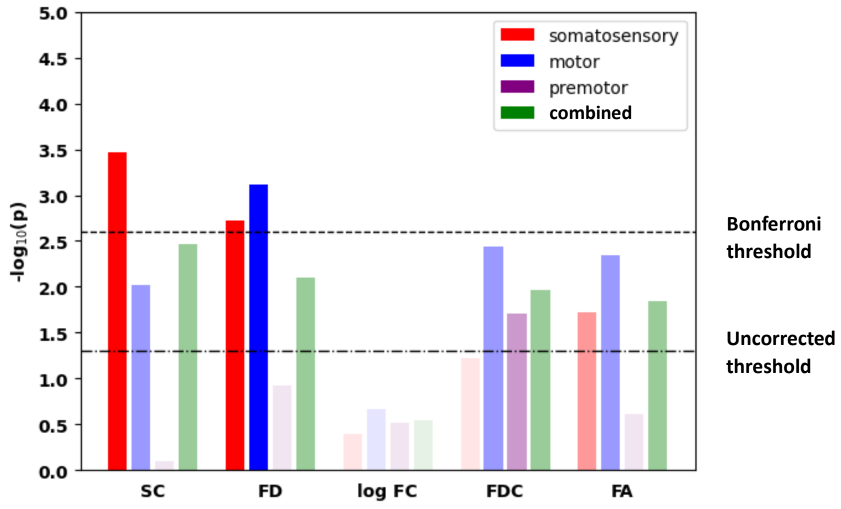

Supplementary Figure 8b. Analyses with matched-sex samples showed very similar results (cf. Figure 2B).
