# Supplementary material for: Quantifying neurodegeneration within subdivisions of core motor pathways in amyotrophic lateral sclerosis using diffusion MRI
Source: J Neurol. 2025 Feb 19;272(3):215. doi: 10.1007/s00415-025-12920-9 (PMC11839792; doi:10.1007/s00415-025-12920-9)
Supplement: Supplementary file 2 — Supplementary file2 (PDF 383 KB) [file 415_2025_12920_MOESM2_ESM.pdf]

Supplementary Tables

Supplementary Table 1. Number of excluded outliers:

Corpus callosum:

SC	N outliers	FDC	N outliers
Total subj	5	Total subj	0
SS	2	SS	0
Motor	2	Motor	0
Premotor	1	Premotor	0
Combined	2	Combined	0
FD	N outliers	FA mid	N outliers
Total subj	1	Total subj	2
SS	0	SS	0
Motor	0	Motor	1
Premotor	1	Premotor	1
Combined	0	Combined	0
(log) FC	N outliers	FA bundle	N outliers
Total subj	2	Total subj	3
SS	0	SS	1
Motor	2	Motor	1
Premotor	1	Premotor	2
Combined	2	Combined	2
All metrics	N outliers		
Total subj	11		
SS	3		
Motor	5		
Premotor	5		
Combined	6		

Supplementary Table 1a. *Total subj(ects) depicts the number of subjects that showed outliers in any segment for that metric.*

CST:

SC	N outliers	FDC	N outliers
Total subj	1	Total subj	3
SS	0	SS	2
Motor	1	Motor	0
Premotor	1	Premotor	1
Combined	0	Combined	0
FD	N outliers	FA bundle	N outliers
Total subj	1	Total subj	0
SS	0	SS	0

Motor	0	Motor	0
Premotor	1	Premotor	0
Combined	1	Combined	0
(log) FC	N outliers	All metrics	N outliers
Total subj	2	Total subj	7
SS	2	SS	4
Motor	2	Motor	3
Premotor	2	Premotor	5
Combined	2	Combined	3

Supplementary Table 1b. *Total subj(ects) depicts the number of subjects that showed outliers in any segment for that metric.*

Supplementary Table 2. Statistical details regarding the differences between ALS and controls

CC:

Metric	Segment	Mean ALS	Mean CON	t-val	p-val	BF p-val	Cohen's d
**SC**							
	**SS**	0.55	0.66	-1.82	0.07	1.0	0.41
	**Motor**	**7.67**	**11.58**	-3.86	< 0.001	**0.008***	**0.96**
	**Premotor**	11.89	14.08	-2.31	0.02	0.587	0.45
	**Combined**	**30.97**	**41.05**	-3.77	< 0.001	**0.009***	**0.89**
**FD**							
	**SS**	**0.43**	**0.47**	-3.77	< 0.001	**0.010***	**0.85**
	**Motor**	**0.51**	**0.57**	-4.72	< 0.0001	**< 0.001***	**1.13**
	**Premotor**	0.49	0.53	-3.01	< 0.01	0.092	0.63
	**Combined**	**0.47**	**0.51**	-4.02	< 0.001	**0.004***	**0.90**
**(log) FC**							
	**SS**	-0.04	-0.05	0.30	0.77	1.0	-0.08
	**Motor**	-0.02	-0.04	0.93	0.36	1.0	-0.29
	**Premotor**	-0.01	-0.04	0.91	0.36	1.0	-0.28
	**Combined**	-0.02	-0.05	0.94	0.35	1.0	-0.29
**FDC**							
	**SS**	0.41	0.44	-2.12	0.04	0.924	0.49
	**Motor**	0.50	0.54	-2.91	< 0.01	0.123	0.67
	**premotor**	0.49	0.51	-1.54	0.13	1.0	0.29
	**Combined**	0.46	0.49	-2.32	0.02	0.571	0.50
**FA mid**							
	**SS**	0.66	0.69	-0.96	0.34	1.0	0.29
	**motor**	0.68	0.72	-2.51	0.01	0.357	0.72
	**premotor**	0.71	0.73	-1.81	0.08	1.0	0.51
	**Combined**	0.69	0.72	-2.57	0.01	0.296	0.73
**FA bundle**							
	**SS**	0.50	0.51	-0.47	0.64	1.0	0.13
	**motor**	0.52	0.54	-2.98	< 0.01	0.100	0.79
	**premotor**	0.53	0.53	-0.63	0.53	1.0	0.11

	Combined	0.52	0.53	-1.94	0.06	1.0	0.48

*Supplementary Table 2a. SS = somatosensory. BF = Bonferroni corrected (i.e., p-value*24).*

CST:

Metric	Segment	Mean ALS	Mean CON	t-val	p-val	BF p-val	Cohen's d
**SC**							
	**SS**	6.12	8.02	-3.63	< 0.001	**0.012***	**0.79**
	**Motor**	13.48	15.98	-2.62	0.01	0.218	0.74
	**Premotor**	7.67	7.56	-0.05	0.96	1.0	-0.04
	**Combined**	27.70	31.56	-2.50	0.01	0.297	0.64
**FD**							
	**SS**	0.50	0.53	-3.43	< 0.01	**0.022***	**0.87**
	**Motor**	0.54	0.57	-3.53	< 0.001	**0.016***	**0.94**
	**Premotor**	0.52	0.53	-1.75	0.08	1.0	0.47
	**Combined**	0.49	0.501	-2.79	< 0.01	0.139	0.71
**(log) FC**							
	**SS**	-0.04	-0.05	-0.13	0.90	1.0	-0.01
	**Motor**	-0.04	-0.03	-0.48	0.63	1.0	0.04
	**Premotor**	-0.02	-0.03	-0.14	0.89	1.0	-0.04
	**Combined**	-0.03	-0.03	-0.24	0.81	1.0	-0.00
**FDC**							
	**SS**	0.48	0.49	-1.29	0.20	1.0	0.28
	**Motor**	0.52	0.55	-2.42	0.02	0.362	0.57
	**premotor**	0.50	0.52	-1.69	0.10	1.0	0.36
	**Combined**	0.46	0.49	-1.93	0.06	1.0	0.43
**FA bundle**							
	**SS**	0.53	0.55	-2.12	0.05	0.750	0.57
	**motor**	0.53	0.56	-2.70	< 0.01	0.178	0.76
	**premotor**	0.53	0.53	-0.95	0.34	1.0	0.24
	**Combined**	0.53	0.55	-2.23	0.03	0.587	0.62

*Supplementary Table 2b. SS = somatosensory. BF = Bonferroni corrected (i.e., p-value*20).*

Supplementary Table 3. Statistical details regarding the association with ALSFRS

CC:

Metric	Segment	t-val	p-val	BF p-val	Pearson's r
**SC**					
	**SS**	1.31	0.20	1.0	0.20
	**Motor**	1.70	0.09	1.0	0.24
	**Premotor**	0.79	0.44	1.0	0.11
	**Combined**	1.72	0.09	1.0	0.22
**FD**					
	**SS**	2.30	0.03	0.627	0.32
	**Motor**	2.06	0.04	1.0	0.29
	**Premotor**	1.52	0.14	1.0	0.23
	**Combined**	1.71	0.09	1.0	0.25
**(log) FC**					
	**SS**	3.46	< 0.01	**0.030***	**0.46**
	**Motor**	3.59	< 0.001	**0.019***	**0.48**
	**Premotor**	2.45	0.03	0.452	0.35
	**Combined**	3.06	< 0.01	0.090	0.42
**FDC**					
	**SS**	4.38	< 0.0001	**0.001***	**0.54**
	**Motor**	4.39	< 0.0001	**0.001***	**0.52**
	**premotor**	2.62	0.01	0.288	0.35
	**Combined**	4.04	< 0.001	**0.004***	**0.49**
**FA mid**					
	**SS**	1.29	0.20	1.0	0.19
	**motor**	2.61	0.01	0.287	**0.37**
	**premotor**	2.20	0.03	0.806	0.32
	**Combined**	2.47	0.02	0.431	0.35
**FA bundle**					
	**SS**	2.03	0.05	1.0	0.29
	**motor**	3.32	< 0.01	**0.043***	**0.45**
	**premotor**	1.66	0.10	1.0	0.24
	**Combined**	2.62	0.01	0.292	0.36

Supplementary Table 3a. SS = somatosensory. BF = Bonferroni corrected (i.e., p-value*24).

CST:

Metric	Segment	t-val	p-val	BF p-val	Pearson's r
**SC**					
	**SS**	1.47	0.15	1.0	0.21
	**Motor**	1.69	0.099	1.0	0.25
	**Premotor**	1.94	0.058	1.0	0.28
	**Combined**	1.91	0.063	1.0	0.28
**FD**					
	**SS**	2.55	0.014	0.286	0.36
	**Motor**	2.53	0.015	0.294	0.36
	**Premotor**	1.34	0.185	1.0	0.2
	**Combined**	1.61	0.114	1.0	0.23
**(log) FC**					
	**SS**	3.6	< 0.001	**0.016***	**0.48**
	**Motor**	4.05	< 0.001	**0.004***	**0.50**
	**Premotor**	2.78	0.008	0.167	0.38
	**Combined**	3.45	0.001	**0.024***	**0.46**
**FDC**					
	**SS**	5.18	< 0.0001	**< 0.001***	**0.60**
	**Motor**	5.13	< 0.0001	**< 0.001***	**0.59**
	**premotor**	4.0	< 0.001	**0.005***	**0.50**
	**Combined**	4.66	< 0.001	**< 0.001***	**0.56**
**FA bundle**					
	**SS**	2.55	0.014	0.280	0.36
	**motor**	3.03	0.004	0.080	0.42
	**premotor**	3.05	0.004	0.078	0.42
	**Combined**	3.01	0.004	0.084	0.42

Supplementary Table 3b. SS = somatosensory. BF = Bonferroni corrected (i.e., p-value*20).

Supplementary Table 4. Longitudinal changes.

CC:

Metric	Segment	Mean (SD) change*	t-val	p-val
**SC**				
	**Motor**	-0.81 (1.69)	-1.18	0.38
**FD**				
	**Motor**	-0.01 (0.02)	-1.32	0.23
**(log) FC**				
	**Motor**	-0.02 (0.02)	**-2.88**	**0.03***
**FDC**				
	**Motor**	-0.02 (0.02)	-2.01	0.11
**FA mid**				
	**Motor**	-0.01 (0.03)	-1.23	0.30
**FA bundle**				
	**Motor**	-0.01 (0.01)	**-2.47**	**0.05***

*Supplementary Table 4a. *Follow-up minus baseline.*

CST:

Metric	Segment	Mean (SD) change*	t-val	p-val
**SC**				
	**Motor**	-1.71 (2.05)	-2.04	0.13
**FD**				
	**Motor**	-0.01 (0.02)	-0.82	0.43
**(log) FC**				
	**Motor**	-0.02 (0.02)	-2.69	0.07
**FDC**				
	**Motor**	-0.01 (0.02)	-1.94	0.11
**FA bundle**				
	**Motor**	-0.01 (0.01)	-1.54	0.22

*Supplementary Table 4b. *Follow-up minus baseline.*
